# Atypical presentation of cardiac tamponade in pulmonary hypertension: A case report and review of the literature

**DOI:** 10.1002/ccr3.5218

**Published:** 2021-12-13

**Authors:** Zahra Ansari Aval, Seyed Mohsen Mirhosseini, Sepideh Jafari Naeini

**Affiliations:** ^1^ Cardiovascular Research Center Department of Cardiovascular Surgery Shahid Beheshti University of Medical Sciences Tehran Iran; ^2^ Cardiovascular Research Center Shahid Beheshti University of Medical Sciences Tehran Iran

**Keywords:** cardiac tamponade, pulmonary hypertension, systemic sclerosis

## Abstract

A young woman with systemic sclerosis, hypothyroidism, and pulmonary hypertension was admitted to our center with massive pericardial effusion and left ventricular collapse. Despite undergoing successful pericardiocentesis, she passed away a month later. The best therapeutic approach in this situation remains to be determined.

## Graphical abstract

The optimal method (medical or invasive approach) to treat the presence of concomitant cardiac tamponade and pulmonary hypertension is still unknown. Pericardiocentesis in this group of patients is associated with a high risk of mortality even after discharge.

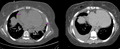

## 1 CASE PRESENTATION

A 32‐year‐old woman with a 7‐year history of systemic sclerosis (SSc), poorly controlled hypothyroidism, and precapillary pulmonary hypertension (pulmonary arterial hypertension (PAH) based on previous right heart catheterization results) was admitted with severe dyspnea, edema, and ascites indicative of right‐sided heart failure. On admission, she was mildly confused with severe hypoxia (oxygen saturation was 75% in room air and 80% with a non‐rebreather mask), low blood pressure (80/55 mm Hg), and sinus tachycardia (120 beats per minute) without fever (T = 36.8°C). On physical examination, she was in a sitting position with distended jugular veins, bilaterally reduced breathing sounds in the basilar parts of both lungs and scattered fine crackles, tachycardia with reduced heart sounds, severe ascites, and bilateral 3+ edema in the lower extremities. She had been poorly followed up while taking sildenafil (50 mg twice daily), bosentan (125 mg twice daily), digoxin, furosemide, levothyroxine, warfarin, and prednisolone. She was under continuous oxygen therapy at home.

Laboratory tests revealed the following results: thyroid‐stimulating hormone (TSH) = 60 mIU/ml, T3 = 0.5 ng/ml, T4 = 3.4 microgram/dl, total bilirubin = 4.5 mg/dl, direct bilirubin = 2.9 mg/dl, urea = 48 mg/dl, creatinine level = 1.24 mg/dl, aspartate transaminase (AST) = 232 μ/L, alanine transaminase (ALT) = 90 μ/L, and N‐type probrain natriuretic peptide (NT Pro BNP) = 19,600 pg/ml.

**FIGURE 1:**
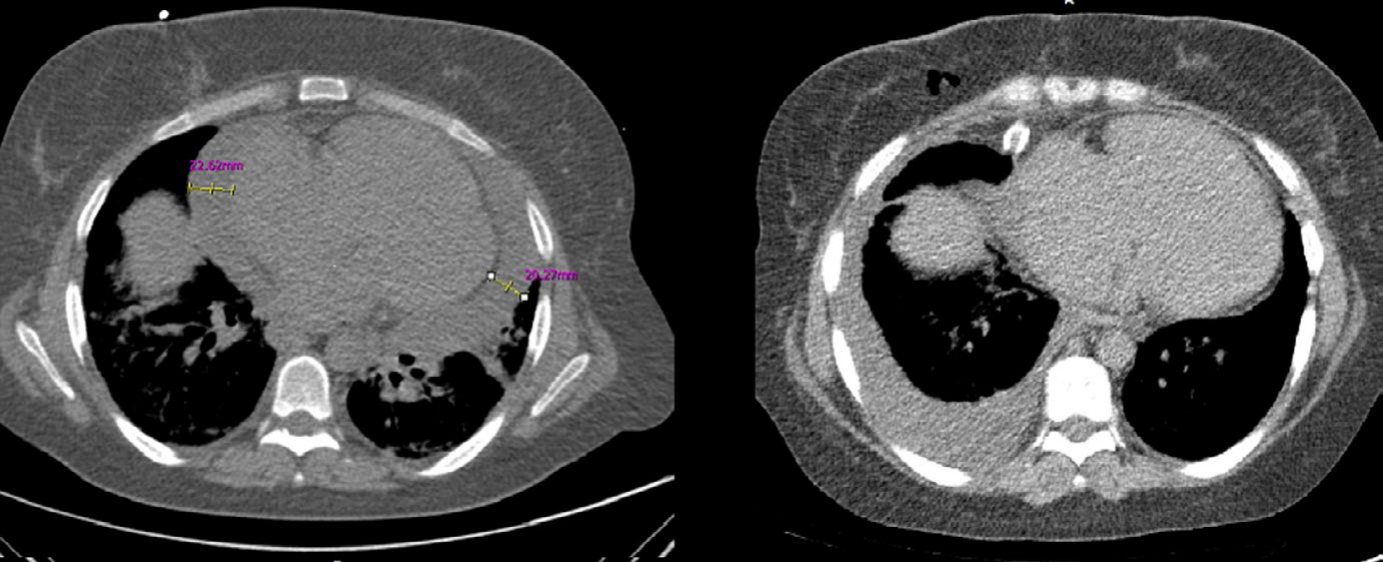
Spiral chest CT scan. Left side: before surgical pericardiocentesis: massive circumferential pericardiocentesis maximum size posterior to LV 25 mm and around RA 24 mm. Right side: after pericardiocentesis: minimal residual pericardial effusion. Abbreviations: CT, Computed tomography; LV, left ventricle; RA, right atrium

Initial bedside transthoracic echocardiography during patient distress revealed normal left ventricular (LV) size and systolic function (LV ejection fraction (EF)>55%), a D‐shaped LV due to severe right ventricular (RV) pressure overload, severe RV enlargement and dysfunction, moderate to severe tricuspid regurgitation with estimated systolic pulmonary artery pressure = 80 mm Hg with massive pericardial effusion (PE), mostly around the LV (maximum size 25 mm posterior to LV), and a small effusion around the LV and RV apex with left atrial (LA) invagination and LV diastolic collapse without RV collapse. A dilated inferior vena cava without collapse was also reported. Evidence of massive pericardial effusion was confirmed with spiral chest computed tomography (CT scan) (Figure 1) due to the poor quality of echocardiography in the semisitting position. ECG showed low‐voltage QRS complexes with sinus rhythm and nonspecific ST‐T changes.

Based on the hemodynamic status of the patient, cardiac tamponade was considered as the most probable diagnosis and after the insertion of an arterial line and a central venous catheter (central venous pressure (CVP) = 25 mm Hg) and initial stabilization with a vasoconstrictor (norepinephrine), she was selected for percutaneous or surgical pericardiocentesis. Based on echocardiography and chest CT scan findings, the effusion around the RV was not significant enough to warrant percutaneous drainage. Thus, she was transferred to an operating room to undergo surgical pericardiocentesis. The procedure was performed via a small subxiphoid incision (about 4 cm). After retracting the xiphoid process and peripericardial fat and making an incision on the pericardium, a pericardial effusion amounting to 400 cc was drained under general anesthesia without complications on the first day with approximately 2 L of fluid drainage on subsequent days using a chest tube (28 french). The effusion was analyzed for bacterial infections, tuberculosis, and malignancy by getting a smear sample, carrying out bacterial culture and measuring the level of adenosine deaminase (ADA). The tests yielded negative results. The patient was extubated the next morning with an acceptable hemodynamic profile. She was completely alert with no signs of hypoperfusion. A supportive inodilator (milrinone) and norepinephrine were continued for 2 weeks with intravenous prostaglandin E1, alprostadil (the only available form), which was administered for a week, and she was discharged with oral medications to treat pulmonary hypertension (sildenafil 50 mg three times daily and bosentan 125 mg twice daily). Based on an endocrinology consultation, her dosage of levothyroxine was increased. She was advised to follow weekly outpatient prostaglandin infusions. Unfortunately, the clinical symptoms of RV failure appeared soon after discharge (exacerbation of edema, ascites, and dyspnea), but the patient refused to be readmitted and passed away a month later.

## 2 DISCUSSION

As an inflammatory autoimmune disorder with diffuse involvement of various organs, systemic sclerosis can be manifested by pericardial effusion. The prevalence of clinically symptomatic pericardial effusion (PE) in SSc is 5%–16%.^1^ On the other hand, the prevalence of pulmonary artery hypertension (PAH) in SSc (systemic sclerosis‐induced PAH (SSc‐PAH)) has been reported to be around 10%–15%, with a higher prevalence in limited cutaneous type.^2^ Some studies have concluded that pulmonary hypertension in systemic sclerosis is associated with a poor prognosis. Vascular endothelial damage, inflammation, and fibrosis are possible mechanisms involved in the pathogenesis of SSc‐PAH.^2^

Cardiac tamponade is reported in about 0.02% of PAH cases.^3^ Even in the absence of tamponade, based on pulmonary hypertension (PH) guidelines, pericardial effusion in the presence of PH is basically indicative of a high‐risk patient.^4^ Pulmonary hypertension may lead to right ventricular failure as a known cause of pericardial effusion in systemic sclerosis.

In this setting, high right atrial (RA) and RV pressures are considered as causes of obstruction in the venous and lymphatic system and cytokine release, which can lead to pericardial effusion.^3^, ^5^, ^6^ It can also result in an increase in the pressure of the thebesian vein and the coronary sinus.^6^

Increased intrapericardial pressure in the presence of elevated right‐sided pressures may lead to diastolic LA and LV collapse and low cardiac output^7^ instead of the usual form of RA systolic and RV diastolic collapse, and the classic pattern of tamponade is not expected in the presence of high PAP, RA, and RV pressures.^8^ Our patient had a rare presentation with multiple poor prognostic factors, including pulmonary hypertension and right ventricular failure concomitant with systemic sclerosis and severe hypothyroidism. In addition to SSc, underlying hypothyroidism has been reported as another cause of pulmonary hypertension and pericardial effusion.^9^ Previous studies have reported the association of hypothyroidism and autoimmune diseases like scleroderma and also PAH.^9^ All these factors led to the patient presenting with an atypical form of cardiac tamponade with left‐sided chamber collapse instead of the typical presentation of RA and RV collapses.

In comparison with the usual presentation of tamponade in which fluid drainage, even in low volume, can lead to hemodynamic improvement, in the presence of PAH, sudden drainage of the effusion can be fatal.^5^ Due to the high risk of mortality associated with drainage, even in the presence of tamponade in these cases, there has been a trend toward medical treatment.^1^

Improvement of the hemodynamic status by specific pulmonary hypertension treatments has also been associated with a reduction in the occurrence of pericardial effusion.^7^

The presence of pericardial effusion can protect the RV against the increased afterload induced by PH. After the drainage of the PE, the venous return starts to make an increase in transmural pressure and acute RV dilation may lead to hemodynamic collapse as a result of decreased RV cardiac output and increased pressure in the LV. The residual PE after drainage can continue to support the RV to prevent a drop in cardiac output.^8^

This procedure can also be performed with a Swan‐Ganz catheter and concomitant monitoring of intrapericardial pressure to reduce pericardial pressure to a level below the biatrial diastolic pressures^8^ or, alternatively, under the guidance of echocardiography to limit complications.^6^

## 3 CONCLUSIONS

The optimal approach to pulmonary hypertension‐associated cardiac tamponade is still an issue for debate. It appears that even after successful drainage, the underlying cause is a major determinant of the outcome of the patient. The final decision for drainage should be made by a team of specialists considering different aspects of the disease and patient hemodynamic.

## CONFLICT OF INTEREST

None declared.

## AUTHOR CONTRIBUTIONS

ZAA contributed to data gathering and editing the text. SMM contributed to editing the text. SJN contributed to writing the text and data gathering.

## ETHICAL APPROVAL

Authors have access to all source data for this case report. Preprint version of this paper has been already published on Authorea.com.

## CONSENT

Written informed consent was obtained from the patient's family to publish this report in accordance with the journal's patient consent policy.

## Data Availability

The data that support the findings of this study are available on request from the corresponding author. The data are not publicly available due to privacy or ethical restrictions.
